# Comprehensive analysis of soybean cultivars’ response to SMV infection: genotypic association, molecular characterization, and defense gene expressions

**DOI:** 10.1186/s43141-023-00558-x

**Published:** 2023-10-17

**Authors:** Mohammed A. Eid, Gehan N. Momeh, Abd El-Raheem R. El-Shanshoury, Nanis G. Allam, Reda M. Gaafar

**Affiliations:** Botany and Microbiology Department, Faculty of Science, Tanta University, Tanta, 31527 Egypt

**Keywords:** Soybean mosaic virus, Soybean cultivars, ELISA, PCR, Gene expression, Plant defense

## Abstract

**Background:**

Soybean mosaic virus (SMV) is a devastating disease that threatens soybean plants worldwide. The different soybean genotypes displayed different responses to SMV strains. This study aimed to investigate the response of different selected soybean cultivars to SMV infection in Egypt based on their specific genetic makeup.

**Result:**

The symptoms of SMV infection and the viral concentration were evaluated in eight soybean cultivars (*Giza 21, Giza 22*, *Giza 35*, *Giza 82, Giza 111*, *Crawford, H4L4*, and *PI416937*) using ELISA assay. The results indicated that *Giza 21* and *Giza 35* were moderately tolerant to SMV infection, while *Giza 82* was the least tolerant cultivar. *Giza 22*, *Giza 111*, and *PI416937* were less tolerant; however, *H4L4* and *Crawford* were identified as the most tolerant cultivars against SMV infection. The chi-square analysis showed a significant association between the different selected cultivars and their response against SMV infection. The PCR test showed the presence of *RSV1 (3gG2)*, *RSV1 (5gG3)*, and *RSV3* loci, and the absence of the *RSV4* locus gene. The expression analysis of the selected defense genes *(EDS1*, *PAD4*, *EDR1*, *ERF1*, and *JAR*) showed variations in the fold changes between infected and non-infected soybean cultivars, suggesting that these genes might play a crucial role in this pathosystem. Additionally, there was a strong positive association between the expression levels of *EDR1* and *ERF1*.

**Conclusion:**

The study found the presence of *RSV1 (3gG2)*, *RSV1 (5gG3)*, and *RSV3* loci in selected soybean cultivars, but not *RSV4*. The analysis of gene expression indicated that certain defense genes may play a vital role in the pathosystem. This research is the first of its kind in Egypt to genotype soybean cultivars regarding different *RSV* loci. The findings could be beneficial for further research on understanding the molecular mechanisms involved in SMV infection and its management.

## Background

Soybean (*Glycine max L*.) is one of the most precious legume crops worldwide. It is characterized by high-quality protein and oil for human and animal consumption [1]. However, various biotic and abiotic stresses, such as diseases, pests, drought, and salinity threaten its production and yield [2]. Soybean mosaic virus (SMV) is one of the most devastating diseases, causing significant yield losses and seed quality deterioration. Its infection causes various symptoms in soybean plants, such as mosaic, mottling, necrosis, stunting, vein clearing, and pod malformation, which can reduce yield and seed quality significantly [3]. SMV belongs to the genus *Potyvirus* and the family *Potyviridae* and has a single-stranded positive-sense RNA genome of about 9.6 kb that encodes several proteins involved in replication, movement, and virulence [4]. SMV is transmitted by aphids in a non-persistent manner and by seeds in infected plants. SMV has a narrow natural host range, mainly infecting plants in the family *Fabaceae*, but also some other families such as *Amaranthaceae* and *Solanaceae* [3]. There are several strains of SMV with high genetic diversity based on their virulence on soybean cultivars with different resistance genes [5].

This resistance in soybean depends on genes, which were recognized as Rsv1, Rsv3, and Rsv4, which confer different levels of resistance against the different strains of SMV [5]. The Rsv1 gene is located on chromosome 13 and has multiple alleles that can recognize different SMV strains. The Rsv3 gene is located on chromosome 14 and confers resistance to most SMV strains. The Rsv4 gene is located on chromosome 2 and provides resistance to SMV strains that overcome Rsv1 and Rsv3 [5]. Several studies have documented the relationship of RSV resistance genes and partial defense genes to SMV in soybean cultivars [6–9].

Enhanced disease susceptibility 1 (*EDS1*), phytoalexin deficient 4 (*PAD4*), enhanced disease resistance 1 (*EDR1*), ethylene response factor 1 (*ERF1*), and jasmonic acid-responsive (*JAR*) are key regulators in the plant defense mechanisms against a wide range of pathogens, including viruses. For instance, in *Arabidopsis*, these genes are involved in pathogen-induced programmed cell death, systemic acquired resistance, and the production of phytohormones such as salicylic acid and ethylene, which act as signaling factors that induce the expression of resistance genes [10–13]. *EDS1* is essential for resistance to tobacco mosaic virus (TMV) [14, 15], and *PAD4* contributes to resistance against viruses like potato virus X (PVX) and TMV [16], while EDR1 plays a role in resistance against viruses such as cucumber mosaic virus (CMV) and TMV [16]. ERF1 and JAR have been shown to play a positive role in resistance against viruses such as PVX, TuMV, CMV, and TMV [17]. However, their role in the defense response of soybean against SMV infections and their relationship with Rsv genes remain poorly understood. Understanding the molecular mechanisms underlying the interaction between Rsv genes and the defense-related genes in soybean cultivars is crucial for the development of more resilient soybean varieties with improved resistance against this infection.

In this study, we evaluated different soybean cultivars’ responses to SMV infection using various methods such as symptoms, ELISA assays, chi-square analysis, PCR tests, and gene expression analysis. The research also aimed to investigate the relationship between expression level changes of some selected defense genes *(EDS1*, *PAD4*, *EDR1*, *ERF1*, and *JAR)*, and Rsv1, Rsv3, and Rsv4 loci gene patterns. To the best of our knowledge, this is the initial research conducted in Egypt that involved genotyping soybean cultivars regarding various Rsv loci. In addition, the study’s findings could aid further research into the molecular mechanisms underlying SMV infection and its management.

## Methods

### Soybean cultivars and planting conditions

This study evaluated the response of eight different widely distributed Egyptian soybean cultivars, namely *Giza 21*, *Giza 22*, *Giza 35*, *Giza 82*, *Giza 111*, *Crawford*, *H4L4*, and PI *416937*, to SMV infection. The soybean seeds were obtained from two institutions: the Field Crop Research Institute at SAKHA Agricultural Center, Research Station, Kafr El-Sheikh, and the Agriculture Research Center, Giza. The study was conducted at the greenhouse of the Botany Department, Faculty of Science, Tanta University, Egypt, during the 2020, 2021, and 2022 seasons. The experiments aimed to evaluate the resistance components and defense genes of the soybean cultivars in the open greenhouse using common cultural practices for soybean [18]. The plants were sown in pots and the field was in three replications.

### Soybean mosaic virus (SMV) strain

The SMV isolate utilized in this study was obtained from naturally infected soybean plants. These plants displayed symptoms such as mosaic, yellowing, stunting, leaf curling, and malformation and were collected from different soybean cultivars, *GIZA 111* and *H4L4* [19]. The field where the severely symptomatic soybean cultivars were grown was situated at the Research Station Farm, Agricultural Research Center (ARC), Giza, Egypt, during the growing seasons of 2020 to 2022. The introduced SMV into this field was previously isolated and identified as SbMV using diverse diagnostic techniques, including host reactions, aphid transmission, electron microscopy, serological tests, and RT-PCR [20]. The identified SMV was kindly provided by Dr. Ahmed El-Nabawi from the Plant Viruses Department, Agriculture Research Center, Giza, Egypt.

### Virus mechanical inoculation

The inoculation of different soybean cultivars was done mechanically by using an SMV-infected leaf sample collected from the aforementioned soybean cultivars according to the methods described by Li et al. [21]. Briefly, the inoculum was prepared by homogenizing the fresh symptomatic leaves in a phosphate-buffered saline solution. Carborundum powder was added as an abrasive and the inoculum was applied using a paintbrush or cheesecloth pads to the completely unfolded unifoliolate leaves of the soybean plants [22]. The plants that were not infected were used as controls. The inoculated leaves were rinsed with tap water after the application, and pesticides were sprayed regularly to prevent cross-infection via aphids in the greenhouse. Additionally, the plants were sprayed with an NPK solution; [a fertilizer that contains nitrogen (N), phosphorus (P), and potassium (K)] to promote growth [20].

### Enzyme-linked immunosorbent assay (ELISA)

The leaf samples were collected from the uppermost trifoliolate leaves of the plants. These samples were assayed using an ELISA complete kit (BIOREBA, Switzerland) according to the manufacturer’s protocol. The SMV levels were determined by measuring the absorbance of the samples at a wavelength of 405 nm (OD_405_) using an ELIZA reader (ChroMate®, Awareness Technology, Romer Labs, Inc., USA). A sample was considered positive for SMV if the OD_405_ reading was three or more times greater than that of a healthy plant extract. The test was performed between 30 and 120 min after substrate addition [23].

### Polymerase chain reaction (PCR) analysis

The PCR analysis was utilized to detect the different RSV genes in the selected soybean cultivars using a PCR master mix (Sigma-Aldrich, USA) and primers designed for each gene using the PhytoZome v13 website (Table 1), according to the manufacturer’s control. The PCR reactions were carried out in an iCycler Thermal Cycler (BioRad, Reinach, Switzerland). After the PCR was completed, the resulting products were separated into a 1.0–1.5% agarose gel in × 0.5 TBE and stained with ethidium bromide to visualize the amplified DNA fragments under a UV lamp [24].

**Table 1:** Primers and gene identifiers of selected genes in *Glycine max L.* genome

**Target primer**	**Gene identifier in *Glycine max L.* genome**	**Primer (5ʹ to 3ʹ)**	**Amplicon sizes**	**Reference**
*Gm RSV1 (5gG3)*	*Glyma.13g190000*	F: CAGCAGCTAGGAGAACTCAATC	501 bp	Original
		R: AGCACCTGTCACACCTTTAC		
*RSV1 (3gG2)*	*Glyma.13g190800*	F: CACTTGCCAGAGCAACTATCT	414 bp	Original
		R: CTCAACATCCACTCCTCCAATC		
*Gm RSV3*	*Glyma.14g204700*	F: CGTTCTCCAGCCTAGTCATTT	315 bp	Original
		R: CACTCCCTTCTTCCACTTCTTC		
*Gm Rsv4*	*Glyma.02g121500*	F: GAACTCTCTGTCCTCTGTGATG	463 bp	Original
		R: GCTCCCTAGTTTCCCATGTT		
*Gm EDS1*	*Glyma.04g177700*	F: GGAGGGTTGCTTGGAGATAA	93 bp	Original
		R: CTTGTCCGTTGACTTGTGATTG		
*Gm PAD4*	*Glyma.08g002100*	F: AGTCCTCTGGCATAGCAAAC	96 bp	Original
		R: CATTCAGGGTTGGTGAAGGA		
*Gm EDR1*	*Glyma.10g159200*	F: GCACCAAGGATAGTGCCTTTA	111 bp	Original
		R: CACCATGAGATGGTTGGATAGG		
*Gm ERF1*	*Glyma.20g203700*	F: CAGAGAGTCGCTTAAGGAGATG	96 bp	Original
		R: CTCAAGGAGTGTTTCCTCTTCA		
*Gm JAR1*	*Glyma.16g026900*	F: GGAGCCAGGACACTATGTAATC	125 bp	Original
		R: CTTTGCGAGAGCTGGTGTAA		

### RNA extraction

To extract the total RNA, the High Pure RNA Isolation Kit (miRNeasy, Mini Kit 50, QIAGEN) was utilized following the kit protocol. The extracted RNA from the soybean leaf samples was analyzed for its quantity, purity, and integrity using NanoDrop (Boeco, Germany) by measuring the OD_260/280_. The concentration of all extracted RNA samples was adjusted to the same level using RNAase-free water before the next step.

### Complementary DNA (cDNA) synthesis

The reverse transcriptase reaction was accomplished for the previous preparations using the Transcription First Strand cDNA Synthesis Kit (Roche, Switzerland) according to the kit protocol. The quantity and quality of the synthesized cDNA were assessed using NanoDrop (Boeco, Germany) by measuring the OD_260/280_. The concentration of all synthesized cDNA samples was adjusted to the same level using RNAase-free water before the next step.

### Real-time PCR analysis

The previous five cDNA preparations were analyzed to quantify relative differences in transcript levels. We designed gene-specific primers for five selected defense genes *(EDS1*, *PAD4*, *EDR1*, *ERF1*, and *JAR)* to generate PCR products of less than 200 bp (Table 1). The endogenous tubulin (TUB) transcript level was used to normalize the transcript level of each target gene. We carried out a 20-μL reaction (0.4 mM of each primer, 10 µL of × 2 ready-to-use SYBR Green Master Mix (TaKaRa, Japan), 100 ng of cDNA, and sterile RNAase-free water) using RotorGene Q 5plex (Qiagen, USA). The cycling conditions were: 30 s at 95 °C for preheating and enzyme activation, followed by 40 cycles (melt for 5 s at 95 °C, annealing for 20 s at 60 °C, extension for 45 s at 72 °C, and final extension for 5 min at 72 °C). The differences in relative gene expression were calculated using the 2-log ΔΔCt method [25].

### Statistical analysis

The data entries were recorded in Microsoft Excel (MS Office, 2023, USA) and analyzed using the Statistical Package for the Social Sciences (SPSS v. 26, USA). The quantitative data were presented as means and standard deviations (SDs), and a one-way analysis of variance (ANOVA) was performed to compare continuous variables between two groups of samples, with a significance level set at *p* < 0.05. The qualitative data were presented as numbers and percentages, and the association between variables was determined using the chi-square or Fisher’s exact tests. Multivariate logistic regression analysis was performed on variables with a *p* value less than 0.01 from the univariate analysis. The odds ratios (ORs) were calculated and reported with a 95% confidence interval (CI). A *p* value less than 0.05 was considered statistically significant at 95% CI.

## Results

### Different soybean cultivar’s response to SMV infection

**Fig. 1:**
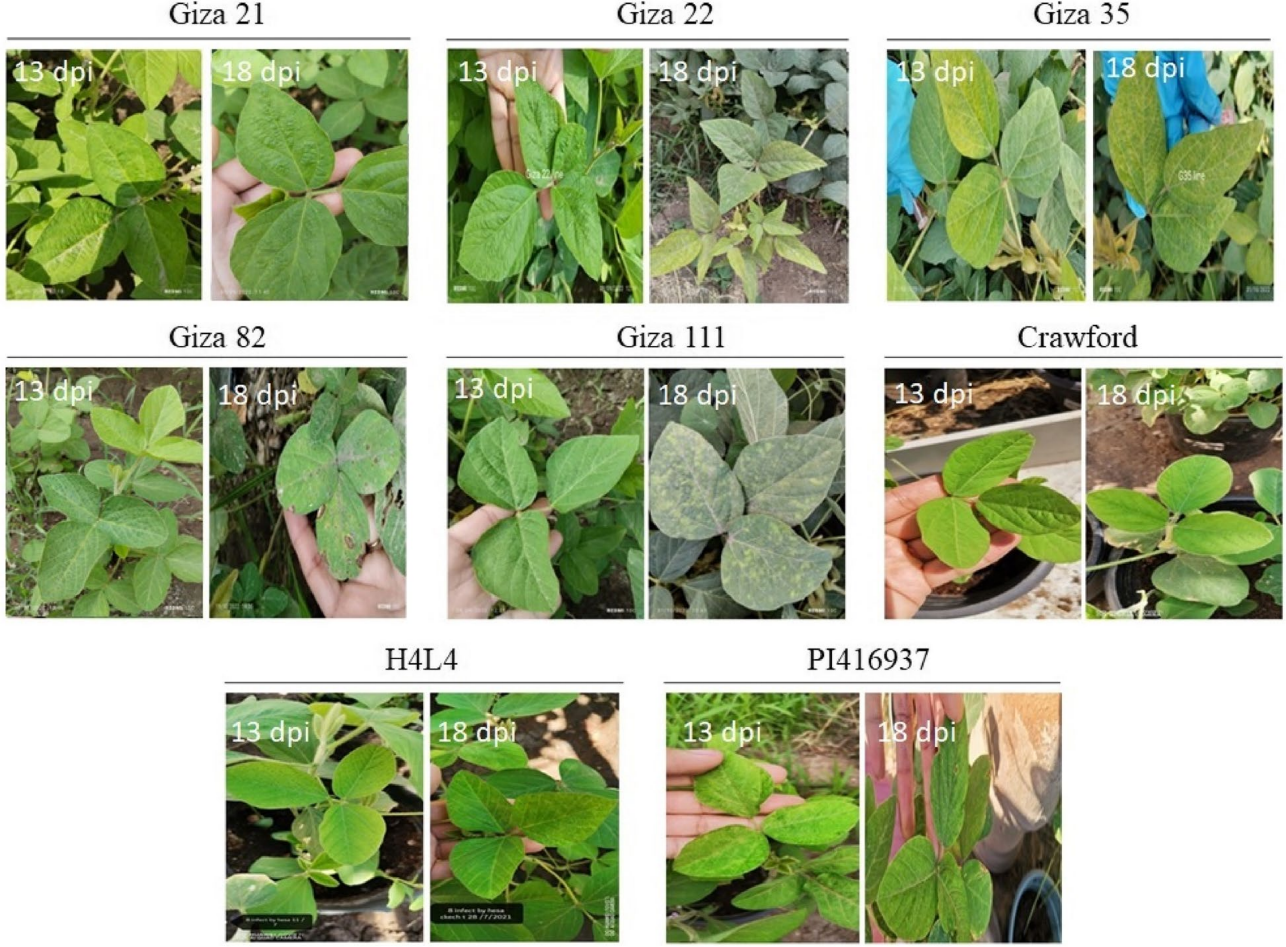
Illustrate the response of eight soybean cultivars to SMV infection. Giza 21 and Giza 35 showed moderate resistance with slight mosaic and mottling at 13 and 18 dpi. Giza 22 and Giza 111 were less resistant exhibiting dark green clusters, and mottling. Giza 82 had the lowest resistance, displaying severe symptoms including vein clearing, mosaic, dwarfing, dark green clusters, and necrosis. PI416937 showed mosaic and dark green clusters. Crawford exhibited slight mosaic at both 13 dpi and 18 dpi. H4L4 had stunted growth, slight mosaic, and systematic necrosis, but only on a few branches. Most soybean cultivars showed stunted growth after SMV infection, and symptoms worsened over time and with other stress factors. H4L4 and Crawford were identified as the most resistant cultivars against SMV infection

The study evaluated the response of eight soybean cultivars (*Giza 21*, *Giza 22*, *Giza 35*, *Giza 82*, *Giza 111*, *Crawford*, *H4L4*, and *PI416937*) to SMV infection. Results indicated that *Giza 21* and *Giza 35* showed moderate resistance to SMV infection as they exhibited slight mosaic and mottling 13 and 18 days after infection (13 and 18 dpi). *Giza 22* and *Giza 111* were less tolerant than *Giza 21* and *Giza 35*, showing mild symptoms of dark green clusters, and mottling. *Giza 82* showed the lowest level of resistance as it displayed higher symptoms of vein clearing, mosaic, dwarfing, dark green clusters along the veins, and necrosis. *PI416937* displayed very low dark green clusters around the veins, and mosaic on the leaves 18 dpi. *Crawford* showed a slight mosaic on the leaves at both 13 and 18 dpi. *H4L4* displayed new leaf stunted growth, slight mosaic, and severe systematic necrosis 18 dpi. However, these symptoms were only observed on a few branches and did not spread to the whole cultivated plant. Most soybean cultivars showed stunted growth after being infected with SMV. The symptoms of all cultivars increased with time and other environmental stress factors. Based on the results, *H4L4* and *Crawford* were identified as the most tolerant cultivar against the used SMV infection (Fig. 1).

### SMV detection in soybean cultivars using an ELISA assay

**Fig. 2:**
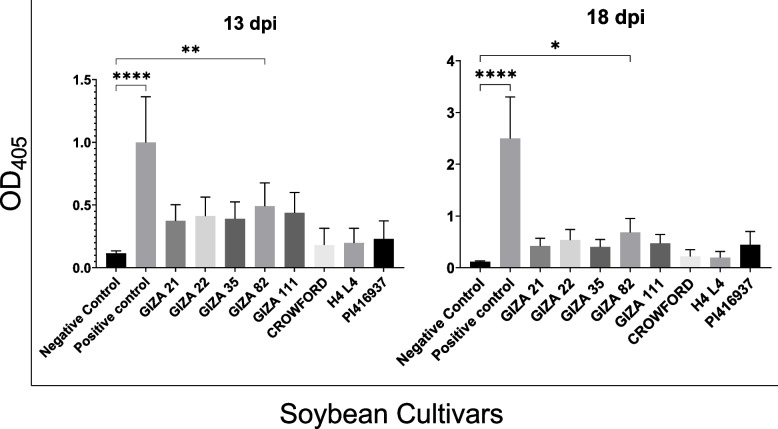
Enzyme-linked immunosorbent assay (ELISA) results for Soybean mosaic virus (SMV) detection in eight soybean cultivars under greenhouse conditions. Asterisks represent a significant difference between the selected groups using one-way ANOVA at *p* < 0.05

An indirect-enzyme-linked immunosorbent assay (ELISA) was used to determine the presence and concentration of SMV in the previously mentioned eight soybean cultivars under greenhouse conditions. The ELISA tests were performed through three seasons (2020, 2021, and 2022) and several times as patches to detect the SMV concentration and presence during the soybean cultivation seasons, especially after 13 to 18 days post-infection. The results in Fig. 2 show that *Giza 82* was significantly the most susceptible cultivar in all seasons, compared to the negative control (non-inoculated soybeans), with OD_405_ readings of 0.49 and 0.68, at 13 dpi, and 18 dpi, respectively, followed by *Giza 22* and *Giza 11*. *Giza 21*, *Giza 35*, *Crawford*, *H4L4*, and *PI416937* were the least susceptible cultivars in all seasons. Consistent with the previous symptom’s description of SMV infection, *Crawford* and *H4L4* showed the lowest viral concentration with OD_405_ of 0.18 at 13 dpi and 0.22 at 18 dpi, and 0.19 at 13 dpi and 0.2 at 18 dpi, respectively, compared to the remaining cultivars.

### Association between different soybean cultivars and their SMV infection response

The chi-square analysis was used to determine if there is a significant association between different tested soybean cultivars and the ELIZA reading of SMV coat protein, which indicates the response of each cultivar against SMV infection (Table 2). The Pearson chi-square value obtained from the analysis is 90 with a degree of freedom (df) of 81.231 and an asymptotic significance (2-sided) value of 0.999. This indicates that there is a significant association between different cultivars and their response to SMV infection, as the *p* value is less than 0.05. Additionally, the Linear-by-Linear Association test yielded a value of 1.2661 with a significance level of 0.260, indicating that there may be a linear relationship between these cultivars and SMV infection.

**Table 2:** The relationship between different soybean genotypes and response to SMV infection through chi-square analysis of ELISA readings

**Soybean cultivars**	**ELIZA reading 13 dpi (OD**_**405**_**)** **Mean ± SD**	**ELIZA reading 18 dpi (OD**_**405**_**)** **Mean ± SD**
Negative control	0.1146 ± 0.02	0.1162 ± 0.01
Positive control	0.9989 ± 0.36	2.501 ± 0.79
*GIZA21*	0.3745 ± 0.12	0.4225 ± 0.14
*GIZA22*	0.4128 ± 0.15	0.5377 ± 0.2
*GIZA35*	0.3905 ± 0.13	0.3997 ± 0.14
*GIZA82*	0.4913 ± 0.18	0.6843 ± 0.26
*GIZA111*	0.439 ± 0.16	0.4695 ± 0.17
*CROWFORD*	0.181 ± 0.13	0.2223 ± 0.12
*H4L4*	0.1969 ± 0.11	0.1969 ± 0.11
*PI416937*	0.229 ± 0.14	0.445 ± 0.25
Pearson chi-square	90.000^a^	df 81, A. S. (2-sided) = 0.231
Likelihood ratio	46.052	df 81, A.S. (2-sided) = 0.999
Linear-by-linear association	1.266	df 1, A. S. (2-sided) = 0.260
*N* of valid cases	10	

The minimum expected count is .10

*SD* Standard deviation, *df* Degree of freedom, *A. S.* Asymptotic significance (2-sided)

^a^100 cells (100.0%) have an expected count of less than 5

### PCR characterization of the soybean cultivars after SMV infection

**Fig. 3:**
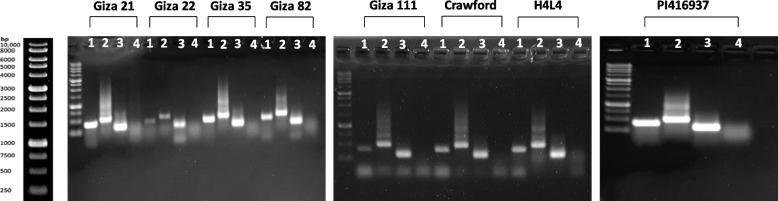
Agarose gel electrophoresis (1.5% agarose) of PCR amplified products using specific primers for each Rsv loci (lane no. 1, RSV1 (3gG2); lane no. 2, RSV1 (5gG3); lane no. 3, RSV3; and lane no. 4, RSV4), tested in the represented eight soybean cultivars

The traditional PCR technique, using specific primers, was utilized to amplify different RSV loci sequences, including RSV1 (*3gG2*), RSV1 (*5gG3*), RSV3, and RSV4, to categorize the genotypes of eight selected soybean cultivars: *Giza 21*, *Giza 22*, *Giza 35*, *Giza 82*, *Giza 111*, *Crawford*, *H4L4*, and *PI416937*. The results revealed the same banding patterns for all cultivars. These patterns indicated the presence of RSV1 (*3gG2*), RSV1 (*5gG3*), and RSV3 loci, and the absence of the RSV4 locus of the selected genes (Fig. 3).

### Expression analysis of defense genes in soybean cultivars using qRT-PCR

**Fig. 4:**
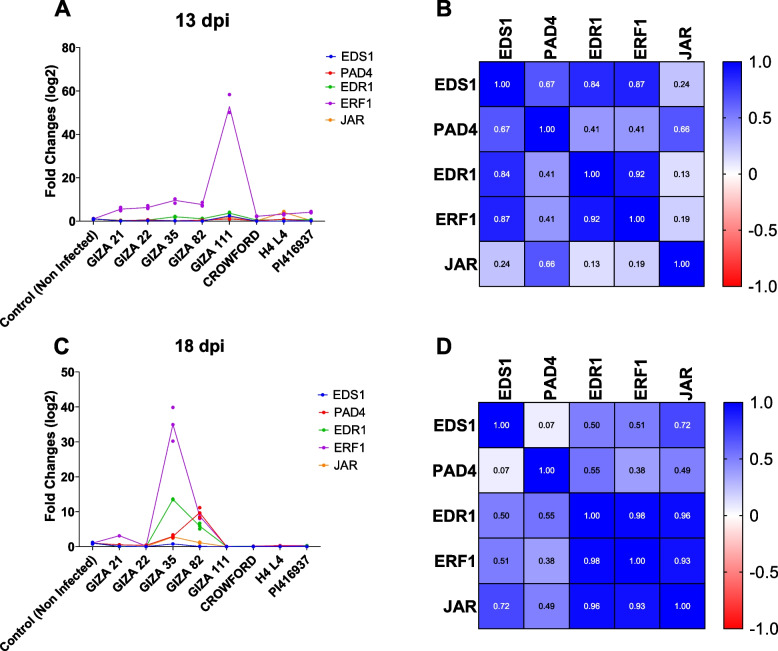
**A**, **C** Fold changes (log2) in gene expression for five different genes (EDS1, PAD4, EDR1, ERF1, and JAR) in both infected and non-infected soybean cultivars at two different time points (13 dpi and 18 dpi). **B**, **D** Pearson correlation analysis revealed a significant positive correlation between the expression level of ERF1 and EDR1 in both 13 dpi and 18 dpi

The data presented in Fig. 4 displays the fold changes (log2) in gene expression for five different genes (*EDS1, PAD4, EDR1, ERF1*, and *JAR*) in both infected and non-infected soybean cultivars at two-time points (13 dpi and 18 dpi). The results demonstrate varying fold changes for these genes in infected versus non-infected soybean cultivars at both time points. For instance, *EDS1* and *JAR* exhibit lower fold changes in infected plants compared to non-infected plants at 13 dpi and 18 dpi, suggesting potential downregulation during infection. Similarly, the fold change for *PAD4* is lower in infected plants at 13 dpi but upregulated at 18 dpi, implying a role in plant defense at later infection stages. *EDR1* and *ERF1* display similar fold changes between infected and non-infected plants at both time points, except for *Giza 35*, *Giza 82*, and *Giza 111*, which exhibit variable upregulation (Fig. 4A, C). Pearson correlation analysis reveals a significant positive correlation between the expression levels of *ERF1* and *EDR1* at both time points, suggesting a functional relationship in the tolerance of selected soybean cultivars against SMV infection (Fig. 4B, D).

### Relationship between soybean cultivars and expression of selected defense genes

Our study explores the connection between different soybean cultivars and the expression of the selected defense genes (*EDS1*, *PAD4*, *EDR1*, *ERF1*, and *JAR*) in response to SMV infection. Multivariable logistic regression analysis investigates relationships among variables (days post-infection, gene expression levels) (Table 3).

**Table 3:** Multivariable regression analysis of soybean cultivars and gene expression of some selected defense genes

**Soybean cultivars and different genes expression**	**(B)** **Unstandardized regression weight**	**(Wald)** **Chi-square distribution**	**(Exp, B)** **Odds ratios**
Control			
Intercept	0.000	0.000	
dpi	0.000	0.000	1.000
*EDS1*	19.002	0.000	178,761,151.770
*PAD4*	−19.002	0.000	5.594E−9
*GIZA21*			
Intercept	0.000	0.000	
dpi	0.000	0.000	1.000
*EDS1*	19.002	0.000	178,761,151.770
*PAD4*	−19.002	0.000	5.594E−9
*GIZA22*			
Intercept	0.000	0.000	178,761,151.770
dpi	0.000	0.000	1.000
*EDS1*	19.002	0.000	178,761,151.770
*PAD4*	−19.002	0.000	5.594E−9
*GIZA35*			
Intercept	0.000	0.000	
dpi	0.000	0.000	1.000
*EDS1*	19.002	0.000	178,761,151.770
*PAD4*	−19.002	0.000	5.594E−9
*GIZA82*			
Intercept	0.000	0.000	
dpi	0.000	0.000	1.000
*EDS1*	−19.002	0.000	5.594E−9
*PAD4*	−19.002	0.000	5.594E−9
*GIZA111*			
Intercept	19.002	0.000	
dpi	0.000	0.000	1.000
*EDS1*	−38.003	0.000	3.129E−17
*PAD4*	−19.002	0.000	5.594E−9
*CROWFORD*			
Intercept	0.000	0.000	
dpi	0.000	0.000	1.000
*EDS1*	−19.002	0.000	5.594E−9
*PAD4*	19.002	0.000	178,761,151.770
*H4L4*			
Intercept	0.000	0.000	
dpi	0.000	0.000	1.000
*EDS1*	19.002	0.000	178,761,151.770
*PAD4*	−19.002	0.000	5.594E−9

The reference category is PI416937

Results show an unstandardized regression weight of 0.000 for dpi, indicating no significant association between days post-infection and soybean resistance to SMV infection. Notably, *EDS1* exhibits a significant positive association with most soybean cultivars, as indicated by an unstandardized regression weight of 19.002. Conversely, *PAD4* shows a significant negative association, with an unstandardized regression weight of −19.002.

Upon individual cultivar analysis, consistent associations emerge between all cultivars and *EDS1/PAD4* gene expression, except for *Giza 82* and *Giza 111*. For *Giza 82*, the unstandardized regression weight for *EDS1* and *PAD4* is −19.002, reflecting a significant negative association. *Giza 111* similarly demonstrates negative associations, with unstandardized regression weights of −38.003 for *EDS1* and −19.002 for *PAD4*. Odds ratios align with overall results.

Chi-square analysis yields a value of 0.000, indicating a strong association between variables and gene expression. Odds ratios for *EDS1* and *PAD4*, at 1.7876115177 × 10^8 and 5.594 × 10^ − 9 respectively, underscore the importance of these genes in soybean resistance to SMV infection.

Overall, these findings suggest that *EDS1* and *PAD4* are important factors for resistance in selected soybean cultivars against SMV infection. Future research could investigate the mechanisms underlying the observed associations and explore the potential applications of these findings in soybean breeding and the development of resistant cultivars.

## Discussion

This study aimed to identify soybean cultivars that are resistant to SMV under greenhouse conditions in Egypt. It is also aimed to investigate the genetic basis of this resistance by selecting some candidate defense genes that may be involved in such a pathosystem. Firstly, we evaluated the response of 8 soybean cultivars (*Giza 21*, *Giza 22*, *Giza 35*, *Giza 82*, *Giza 111*, *Crawford*, *H4L4*, and *PI416937*) to SMV infection by visually observing the symptoms and measuring the viral load using the ELISA technique. The obtained findings revealed that *Giza 21* and *Giza 35* showed moderate resistance to SMV infection, while *Giza 82* showed the lowest level of resistance. *Giza 22, Giza 111*, and *PI416937* were less tolerant; however, *H4L4* and *Crawford* were the most tolerant cultivars. These findings are consistent with another study conducted by Bachkar et al. [26] which aimed to evaluate the resistance of soybean cultivars to SMV infection under both natural field and controlled greenhouse conditions. They screened a total of 46 soybean cultivars for their resistance to SMV infection by inoculating them with SMV in natural fields and controlled greenhouse conditions. The cultivars were evaluated for symptoms of SMV infection, including mosaic, mottling, and stunting. They found that the soybean cultivars exhibited varying levels of resistance to SMV infection, with some cultivars showing complete resistance while others were highly susceptible to SMV [26]. This pattern of differentiation between the responses of the different soybean cultivars is because the symptoms induced by SMV depend on many factors, including the host genotype, virus strain, plant age at infection, and environment. That is, even if the same SMV strain was used, different soybean cultivars may have different genotypes that affect their resistance or susceptibility to SMV infection.

Several resistance genes have been identified in soybean, including Rsv1, Rsv3, and Rsv4, which confer resistance to specific strains of SMV. For instance, Rsv1 encodes a nucleotide-binding leucine-rich repeat (NLR) protein that recognizes the viral coat protein of SMV strains G5 and G7, triggering a defense response in the plant that limits viral replication. Rsv3, on the other hand, encodes a membrane-bound receptor-like protein that recognizes the viral P3 protein of SMV strains G5 and G7, resulting in the activation of downstream defense signaling pathways. Rsv4, a gene discovered more recently, also encodes a membrane-bound receptor-like protein that confers resistance to SMV strains G5 and G7 [19].

Alternatively, different SMV strains may have different virulence and infectivity, which could affect how different soybean cultivars respond to infection [3]. For instance, a study conducted by Seo et al. [27] constructed different infectious clones of SMV G7H and G5H strains, and they reported their molecular characteristics and disease reactions on resistant soybean cultivars. SMV-G7H caused systemic mosaic symptoms or lethal systemic hypersensitivity (LSHR) in certain soybean cultivars that are resistant to SMV-G5H, despite sharing 98.9% amino acid sequence homology with G5H. On the other hand, SMV-G5H could not infect soybean cultivars possessing different SMV resistance genes [27]. Another study conducted by Khatabi et al. [28] evaluated North American isolates of SMV for their ability to overcome resistance conferred by the Rsv4 gene, which is a major source of resistance in soybean cultivars. The researchers examined SMV isolates that had previously been reported to overcome the *Rsv4* resistance gene, as well as newly isolated SMV strains. They found that all the tested SMV isolates were able to overcome the *Rsv4* resistance gene, indicating that the resistance was no longer effective against these isolates. The study also identified a region on the SMV genome that appears to play a key role in breaking Rsv4-mediated resistance. Their study concluded that the *Rsv4* gene, which was once considered a highly effective source of resistance against SMV, has now been rendered ineffective due to the evolution of new SMV strains capable of overcoming it and suggested that new sources of resistance need to be identified to combat this virus [28].

To genotype the RSV loci in the selected cultivars used in this study, we used the regular PCR technique with specific primers designed to amplify different RSV loci sequences. The results revealed that all eight cultivars showed the same banding patterns for RSV1 (3gG2), RSV1 (5gG3), and RSV3 loci, indicating that they share the same alleles for these loci. However, none of the cultivars showed a band for the RSV4 locus, suggesting that they lack this locus or have a different allele that is not detected by the primer pair used. The absence of variation in RSV1 (3gG2), RSV1 (5gG3), and RSV3 loci among the eight cultivars implies that these loci are highly conserved in soybean and may not be useful for distinguishing SMV resistance levels in the selected cultivars. On the other hand, the absence of the RSV4 locus in all cultivars indicates that this locus is either rare or deleted in soybean germplasm, or that it has diverged significantly from its ancestor gene sequence used to design the primers. Further studies are needed to confirm the presence or absence of the RSV4 locus in soybean and to explore its role in conferring SMV resistance.

The data presented in Fig. 4 shows the fold changes (log2) in gene expression for five different genes (*EDS1*, *PAD4*, *EDR1*, *ERF1*, and *JAR*) in both infected and non-infected soybean cultivars at two different time points (13 dpi and 18 dpi). The results show that the fold changes for these genes vary between infected and non-infected soybean cultivars at both time points. For example, at 13 dpi and 18 dpi, the fold changes for *EDS1* and *JAR* are lower in infected plants compared to non-infected plants. This suggests that these genes may be downregulated during infection. The fold change for *PAD4* is also lower in infected plants at 13 dpi, but it is upregulated at 18 dpi. This suggests that *PAD4* may play a role in plant defense against infection at later stages of infection. At both time points, the fold changes for *EDR1* and *ERF1* are similar between infected and non-infected plants (Fig. 4A, C). These results suggest that these genes may not play a significant role in plant defense against infection in most selected cultivars except for *Giza 35, Giza 82*, and *Giza 111*, which showed significant upregulation in both time points compared to the other cultivars. In other words, using Pearson correlation analysis the expression level of *ERF1* showed a significant positive correlation with the expression level of *EDR1in* both 13 dpi and 18 dpi (Fig. 4B, D) which might indicate that these two genes may be functionally related to the mediated resistance of tested soybean cultivars against SMV infection.

*EDS1* and *PAD4* are known to interact with each other and function as positive regulators of plant defense responses, including defense against viral infections. The *EDS1-PAD4* complex is required for the activation of SA-induced defense responses. Additionally, the *EDS1-PAD4* complex interacts with several transcription factors, including *ERF1* and *JAR1*, which are known to be involved in the regulation of SA-mediated defense responses [29]. *EDR1*, on the other hand, is a negative regulator of plant defense responses, and its overexpression would lead to increased susceptibility to several plant viruses [30]. *ERF1* is a transcription factor that regulates the expression of genes involved in defense responses against biotic and abiotic stresses. It has been shown to play a role in defense against several plant viruses, including the tobacco mosaic virus and the tomato yellow leaf curl virus [31, 32]. *JAR* is a gene that regulates jasmonate signaling, which is involved in plant defense against herbivores and pathogens. In the study, the authors found that *JAR* was upregulated in soybean plants with extreme resistance to viruses, suggesting that it may play a role in this trait [33].

## Conclusion

This study is the first in Egypt to examine the different cultivars of soybean, based on RSV loci and their response to SMV infection. The findings suggest new roles for the selected genes, particularly in genotypes with the three RSV loci. The overall finding of this study is that the two genes (*ERF1* and *EDR1*) may be functionally related to the moderate resistance of tested soybean cultivars against SMV infection. However, the study is fairly limited in scope, as it only investigates a small number of soybean cultivars and analyzes the expression of only five genes at two-time points. In addition, the specific SMV sequence used in this study was not available, which may have implications for the interpretation of our results. To fully understand soybean defense mechanisms against SMV infection, future studies in this area should prioritize obtaining the full SMV sequence to enable more comprehensive investigations into the specific interactions between viral variants and soybean genotypes. Additionally, exploring alternative molecular techniques, such as next-generation sequencing, may offer opportunities for deeper insights into the genetic basis of soybean resistance to SMV. It would also be beneficial to conduct functional studies to understand the specific roles of the studied genes and to investigate the expression of other genes and pathways involved in plant defense mechanisms against SMV infection in such genotypes. Moreover, including a more diverse range of soybean cultivars in future studies would better represent the soybean population.

## Data Availability

The data generated and/or analyzed in the current study are available from the corresponding authors upon reasonable request.
